# Laryngo-Onycho-Cutaneous Syndrome (LOCS)

**DOI:** 10.12669/pjms.40.2(ICON).9035

**Published:** 2024-01

**Authors:** Fatima Hemani, Uzma Khurram, Anjum Naveed

**Affiliations:** 1 Fatima Hemani Department of Pediatrics, Indus Hospital & Health Network, Karachi, Pakistan; 2 Uzma Khurram, Department of Pediatric Emergency, Indus Hospital & Health Network, Karachi, Pakistan; 3 Anjum Naveed, Department of ENT, South City Hospital, Karachi, Pakistan

**Keywords:** Laryngo-onycho-cutaneous syndrome (LOCS), Shabbir’s syndrome, Junctional Epidermolysis Bullosa (JEB)

## Abstract

Shabbir Syndrome or commonly known as Laryngo-onycho-cutaneous syndrome (LOCS) is an autosomal recessively inherited syndrome, caused due to mutations in the laminin alpha-3 (LAMA3) gene. This syndrome affects the epidermal layer and results in granulation formation in the eyes, larynx, and nails. One of the most dreadful complications of this syndrome can be due to granulation formation in the larynx or sub-glottis region resulting in laryngeal stenosis and death. According to the latest *Online Mendelian Inheritance in Man* (OMIM) classification, LOCS has been reclassified as a subtype of Junctional epidermolysis bullosa (JEB). But it is still considered a rare syndrome with limited cases reported worldwide. In this case report, we have discussed a case of a four year old, Pakistani boy, who presented with stridor, fragile skin, and granulation of nails, with no family history of LOCS.

## INTRODUCTION

Laryngo-onycho-cutaneous syndrome (LOCS), was initially called after the famous Pakistani dermatologist, Dr. Ghulam Shabbir. He first described this syndrome in 1986,^1^ in 22 children, from 12 different families. All these families belonged to a common ethnic group of Punjabi Muslims. LOCS presents as hoarseness of voice, dystrophic changes of nails, deformed teeth, ulcers, and crusting lesions over the face.^2^ These all symptoms are due to excessive granulation tissue formation.^3^ These granulation tissues may lead to early death in children due to laryngeal obstruction. We present a case of a four year old male child, who presented in our emergency room with stridor, nail dystrophy, and ruptured skin lesions, but with no family history of LOCS.

## CASE PRESENTATION

A four year old, male, Punjabi Muslim, was brought to the emergency room by his father at the Indus Hospital and Health Network, Karachi, due to increasing hoarseness of voice. Initially, the child was able to speak normally till the age of two, but then he started experiencing hoarseness, which has now increased to dyspnea. There was no history of fever, tonsillitis, or sinusitis. He has a history of recurrent paronychia and bleeding. He also had multiple small vesicular lesions for the last 1.5 years, mostly focused on ears and nose, which ruptured and bled easily. Also, since the past few weeks, he experienced multiple nose bleeds. Nose bleeds were spontaneous without any history of nose-picking or history of trauma. They were minor bleeds, resolving spontaneously. He had no history of prolonged post circumcision bleeding, hemarthrosis, or easy bruising. He is the 3^rd^ child from a consanguineous marriage, but no other family member including his siblings has any similar complaints.

**Fig.1(a):**
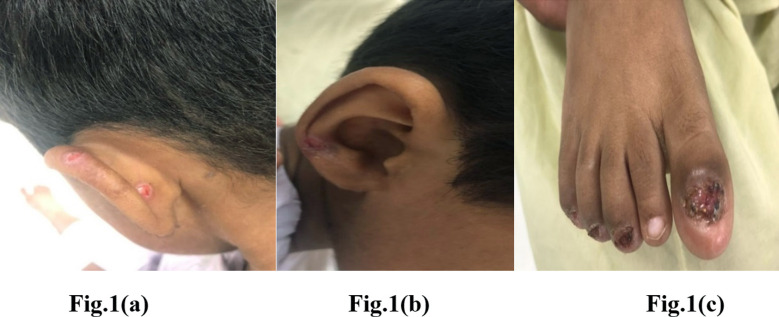
Multiple ruptured hypodense lesions with central pinpoint bleeding. Fig.1(b): One dry crusted lesion over ear lobe. Fig.1(c): Multiple toe nails have fallen off with granulation tissue formation.

On examination, he had a hoarse voice with inspiratory stridor, intermittent sub coastal and suprasternal recessions with bilateral equal air entry. Throat was clear with no apparent obstruction. There were multiple ulcerations on both ears (Fig.1a, 1b) and right nostril, along with erosions of multiple toenails. Multiple nails had fallen off as shown in Fig.1c. No active bleeding, blister, bruising, hemarthrosis, conjunctivitis, deformed teeth, or oral ulcers were observed. The patient was admitted in the Pediatrics unit under Otolaryngology service. His tracheostomy and direct laryngoscopy were done. Tracheostomy was performed under local anesthesia. After tracheostomy, direct laryngoscopy was done under general anesthesia, which showed mucosal growth in glottic and sub glottic region, narrowing the airway. A thick mucosal web was present at the anterior commissure, with normal trachea below. Multiple biopsy samples were taken from the mucosal growth and were send for histopathology assessment. Histopathological evaluation showed fibro-collagenous tissue with ulceration, dense acute and chronic inflammation, granulation tissue formation with separately lying superficial strips of hyperplastic stratified squamous epithelium. Additional investigations performed included complete blood count (CBC), coagulation profile, inflammatory markers and chest x-ray. CBC results showed microcytic anemia along with normal white cell counts.

His histopathological report along with his clinical symptoms confirmed our diagnosis of LOCS. He was discharged after giving instructions to the family regarding tracheostomy care, with plan to follow him in Otolaryngology and Dermatology Consultant Clinic. Dermatology review was performed and he was prescribed topical steroids and fusidic acid cream for his nails. Family was counseled about the disease, inheritance pattern and risk of occurrence in other children.

## DISCUSSION

LOCS , formerly known as Shabbir Syndrome, is a distinct sub type of Junctional epidermolysis bullosae (JEB) according to current classification.^4^ This classification is based on the specific protein laminin α3A been affected in LOCS, causing tissue separation in epidermis.^5^ This presents as granulation formation mostly of eyes, larynx and nails, but may affect other organs too, including dental anomalies like delayed dentation, soft enamel, microdontia and recurrent carries. In the eyes, it presents as bilateral symblepharon recurrent corneal ulceration and scaring, while common skin manifestation includes fragile skin, resulting in easy blistering and ulceration, most commonly affecting cheeks, ears, philtrum, neck and axillary regions. Due to recurrent bleeding episodes in granulating lesions, hypochromic microcytic anemia is commonly seen in LOCS patient at an early age.

Currently three mutations in LAMA 3 gene have been identified as cause of LOCS, among which insertion at exon 39 of LAMA gene is classically seen in Pakistan specific context.^1^,^6^ LOCS was originally considered to be found among Muslim Pakistani Punjabi descends only, but later cases were reported from other parts of the world. So far around 55 cases have been reportedglobally.^7^ Majority of these patients suffer from laryngeal stenosis which results in premature death or tracheostomy.^8^ Literature indicates the occurrence of laryngeal stenosis in LOCS is 80.6%.^6^ Due to limited data availability of the pathophysiology of LOCS, limited treatment options are available. Incision of granulation tissue alone is not a definitive treatment as recurrence of granulation tissue after incision is very frequent. Topical and systemic corticosteroids have been used in some cases for laryngeal stenosis, symblepharon and paronychia, but should be used cautiously in pediatric population due to risk of adrenal suppression.

Our case was different from typical presentation of LOCS, as there was no evidence of autosomal recessive inheritance pattern, as none of the family members was affected previously. A majority of LOCS patients begin experiencing hoarseness at a much younger age.

## CONCLUSION

LOCS is rare syndrome, with limited cases reported so far, many from Pakistan and other Southeast Asian regions. Unfortunately, due to such minimal cases reported limited research has been done to investigate the pathophysiology of this syndrome. It has been establshed that mutations in LAMA3 gene causes defect in the epithelial barrier system, resulting in granulation tissue. But still ample work needs to be done to better understand the mechanisms, for better treatment therapies to prevent fatal complications from occurring in patients with LOCS.
